# Salivary Metabolomics for Prognosis of Oral Squamous Cell Carcinoma

**DOI:** 10.3389/fonc.2021.789248

**Published:** 2022-01-05

**Authors:** Shigeo Ishikawa, Masahiro Sugimoto, Tsuneo Konta, Kenichiro Kitabatake, Shohei Ueda, Kaoru Edamatsu, Naoki Okuyama, Kazuyuki Yusa, Mitsuyoshi Iino

**Affiliations:** ^1^Department of Dentistry, Oral and Maxillofacial Plastic and Reconstructive Surgery, Faculty of Medicine, Yamagata University, Iida-nishi, Japan; ^2^Health Promotion and Pre-emptive Medicine, Research and Development Center for Minimally Invasive Therapies, Tokyo Medical University, Shinjuku, Japan; ^3^Department of Public Health and Hygiene, Yamagata University Graduate School of Medicine, Iida-nishi, Japan

**Keywords:** metabolomics, oral squamous cell carcinoma (OSCC), prognosis, saliva, overall survival, disease-free survival

## Abstract

This study aimed to identify salivary metabolomic biomarkers for predicting the prognosis of oral squamous cell carcinoma (OSCC) based on comprehensive metabolomic analyses. Quantified metabolomics data of unstimulated saliva samples collected from patients with OSCC (n = 72) were randomly divided into the training (n = 35) and validation groups (n = 37). The training data were used to develop a Cox proportional hazards regression model for identifying significant metabolites as prognostic factors for overall survival (OS) and disease-free survival. Moreover, the validation group was used to develop another Cox proportional hazards regression model using the previously identified metabolites. There were no significant between-group differences in the participants’ characteristics, including age, sex, and the median follow-up periods (55 months [range: 3–100] *vs.* 43 months [range: 0–97]). The concentrations of 5-hydroxylysine (p = 0.009) and 3-methylhistidine (p = 0.012) were identified as significant prognostic factors for OS in the training group. Among them, the concentration of 3-methylhistidine was a significant prognostic factor for OS in the validation group (p = 0.048). Our findings revealed that salivary 3-methylhistidine is a prognostic factor for OS in patients with OSCC.

## Introduction

Oral cancer occurs in the oral cavity, with oral squamous cell carcinoma (OSCC) accounting for 90% of all cases of oral cancer (https://gco.iarc.fr/). The oral cavity can be visualized without using special devices; therefore, OSCC is assumed to be easily detected. However, most OSCCs are frequently detected in advanced stages (1, 2), with these OSCCs showing a poor prognosis. Furthermore, there has been no substantial improvement in the long-term survival rate of OSCC in advanced stages over the past few decades (3–6). Therefore, there is a critical need to improve the prognosis of OSCC.

Moreover, it is critical to accurately predict the prognosis of OSCC before oncological treatment. Various clinicopathological parameters can accurately predict the prognosis of OSCC, with cancer staging being the most common predictor (7). An advanced tumor-node-metastasis stage, including cervical lymph node metastasis or distant metastasis, is widely considered to be indicative of a poor prognosis of OSCC (3, 4, 8). Further, the invasion mode and tumor grade are established pathological parameters for predicting prognosis (4). Additionally, the clinical type of tumor growth, including extraversion or inward, is a clinical parameter for predicting the prognosis of oral cancer (4). However, these clinicopathological prognostic parameters should be far from optimal evidence because these predictors have relatively low efficiency and specificity.

Accordingly, molecular biomarkers provide a more objective criterion for prognostic prediction. There is a need for novel strategies to facilitate biomarker-guided treatment selection based on individual tumor differences (2, 9). Recent studies have demonstrated that molecular biomarkers can predict OSCC given the development of analytical methods. Specifically, there has been remarkable development in the application of sequencing technology; moreover, there are numerous ribonucleic acid biomarkers for predicting the prognosis of OSCC (9–11). Additionally, the metabolomic approach to cancer-specific biomarkers is promising. Cancer-specific abnormal metabolism, including the Warburg effect, which utilizes adenosine triphosphate synthesis to sustain rapidly growing cancerous cells rather than readily available oxygen from the surrounding environment, is well described (12, 13). Moreover, salivary metabolomics is an emerging approach for the diagnosis or screening of oral cancers, including OSCC, leukoplakia, and lichen planus (13). Saliva is an ideal biofluid with vast information reflecting the systemic health status that could be used to detect various diseases (12, 13). Applying salivary metabolites is plausible since these molecules may be transferred into saliva by various cells, including OSCC, present in the oral cavity and salivary glands; moreover, saliva allows non-invasive analysis (12). However, to our knowledge, the identification of the prognostic biomarkers of OSCC using salivary metabolomics has not been reported. We aimed to identify salivary metabolomic biomarkers for predicting the prognosis of OSCC.

## Materials and Methods

This study was performed as part of ongoing research on salivary biomarkers for cancer screening at Yamagata University. The study protocol was approved by the Ethics Committee of Yamagata University Faculty of Medicine (#2021-176). All study procedures involving human participants were conducted following the ethical standards of the institutional and/or national research committee, as well as the 1964 Declaration of Helsinki and its later amendments or comparable ethical standards.

Consent was obtained through an online opt-out method, with none of the eligible patients declining participation. Patients with OSCC were recruited from the Department of Dentistry, Oral and Maxillofacial Surgery, Yamagata University Hospital between April 2012 and March 2017. Patients who received curative treatment, such as radical surgery or chemoradiotherapy, were included in this study, whereas patients who received non-curative treatment, such as palliative treatment or symptomatic treatment, were excluded. The total number of patients was 72. One patient rejected surgery and received super-selective intra-arterial chemotherapy and daily concurrent radiotherapy, with the remaining patients undergoing resection surgery. All the patients underwent pathological diagnosis through incisional open biopsy and excised specimens.

### Saliva Collection and Sample Preparation

The protocol for saliva collection has been described previously (13–16). Briefly, before saliva collection, a skilled dentist and dental hygienist checked the oral hygiene of all participants. Remarkable dental plaque and calculus deposits were removed using a toothbrush without dentifrice and ultrasonic scaling at ≥ 3 h before saliva collection. All participants were asked to refrain from eating and drinking for ≥ 1.5 h before saliva collection. The participants rinsed their mouths with water before sample collection and split their saliva into 50 cc Falcon tubes (Corning, Inc., Corning, NY, USA) in a paper cup filled with crushed ice. Subsequently, approximately 3 mL of unstimulated whole saliva was collected for approximately 5 min. Finally, the samples were aliquoted into smaller volumes and stored at -80°C.

### Metabolomic Analysis of Saliva

We performed a metabolomic analysis of saliva samples as previously described (13–17). Briefly, frozen saliva was thawed and dissolved at room temperature. To remove macromolecules, the samples were centrifuged through a 5-kDa cut-off filter (Pall, Tokyo, Japan) at 9100 × g. The filtrate (45 µL) was removed and added to a 1.5-mL Eppendorf tube, followed by the addition and mixing of 5 µL of water containing 2 mM methionine sulphone, 2-(N-morpholino) ethane sulfonic acid, d-camphor-10-sulphonic acid, sodium salt, 3-aminopyrrolidine, and trimesate. Capillary electrophoresis time-of-flight mass spectrometry was performed to quantify the charged metabolites in the positive and negative modes. Raw data were processed using MasterHands software (Keio University, Yamagata, Japan). Metabolites were identified by matching the corresponding m/z values and migration times; further, absolute concentrations were calculated by comparing the peak area (normalized by those of internal standards) with those of standard mixtures (13–17). Our metabolomics data were comprised of two batches of data obtained from 23 (batch 1) and 49 (batch 2) participants, respectively. The data of 20 of the 23 participants in batch 1 were retrieved from a previous study (13), and the data of 3 of the 23 participants in batch 1 were unpublished data. The data of 20 of the 49 participants in batch 2 were retrieved from another previous study (15), and the data of 29 of the 49 participants in batch 2 were unpublished data. Both studies assessed screening of oral cancer using different concepts.

### Statistical Analyses

As aforementioned, we evaluated the unexpected bias caused by two batches. We performed a principal component analysis (PCA) to confirm the between-batch similarity. The distribution of quantitative and qualitative variables was analyzed using the Mann–Whitney U test and chi-square test, respectively. For salivary metabolites, frequently detected metabolites (> 30% of all participants) were used for subsequent analyses. All data (n = 72) were randomly divided into the training (n = 35) and validation (n = 37) groups. Using data from the training group, we calculated the hazard ratios (HRs) and 95% confidence intervals (CIs) using the Cox proportional hazards regression model to assess prognostic factors for overall survival (OS) and disease-free survival (DFS). The multivariate-adjusted model was performed using backward elimination. Significant variables in the multivariate-adjusted model using the training group were included in the Cox proportional hazards regression model using the validation group. Specifically, using significant variables identified from the training group, we calculated HR and 95% CI for assessing the prognostic factors for OS and DFS in the validation group. Regarding the significant variables in the validation group, the survival curves were drawn using the Kaplan-Meier method and compared using the log-rank test. Spearman’s rank correlation coefficient was used to evaluate the relationship between salivary metabolites and continuous variables (age, stage, early phase standard uptake value, and late phase standard uptake value). Furthermore, Mann-Whitney U test was used to evaluate the relationship between salivary metabolites and discrete variable (sex). Statistical analyses were performed using SPSS software, version 20 (SPSS, Inc., Chicago, IL, USA) and MetaboAnalyst (18) (http://www.metaboanalyst.ca/).

## Results

**Figure 1:**
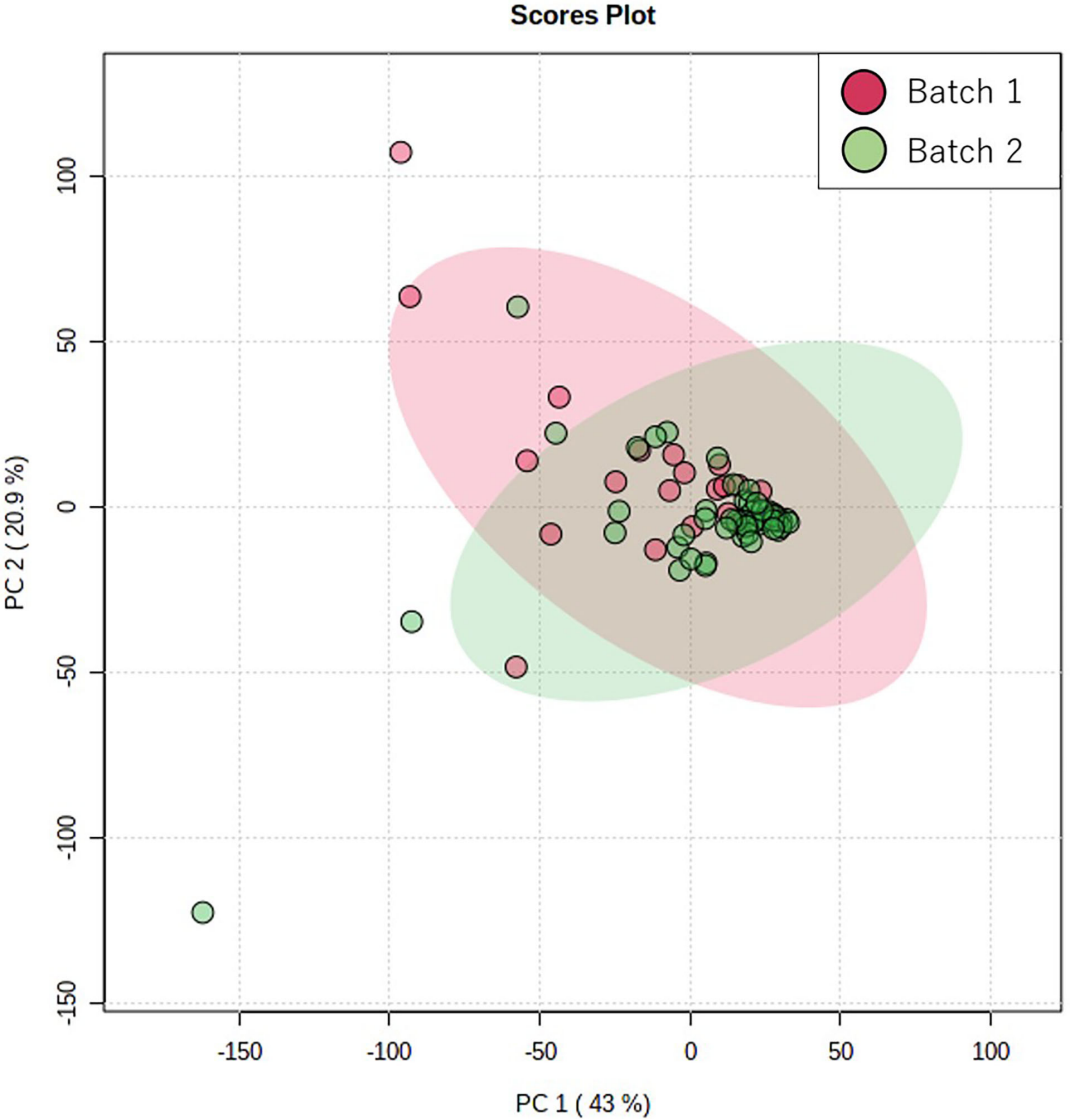
Score plots of principal component analysis using Pareto scaling PC1 and PC2 indicate the first and second principal components, respectively. The red and green plots indicate the samples collected in batches 1 and 2, respectively. The contribution rates of PC1 and PC2 were 43.1% and 20.95%, respectively.

**Figure 2:**
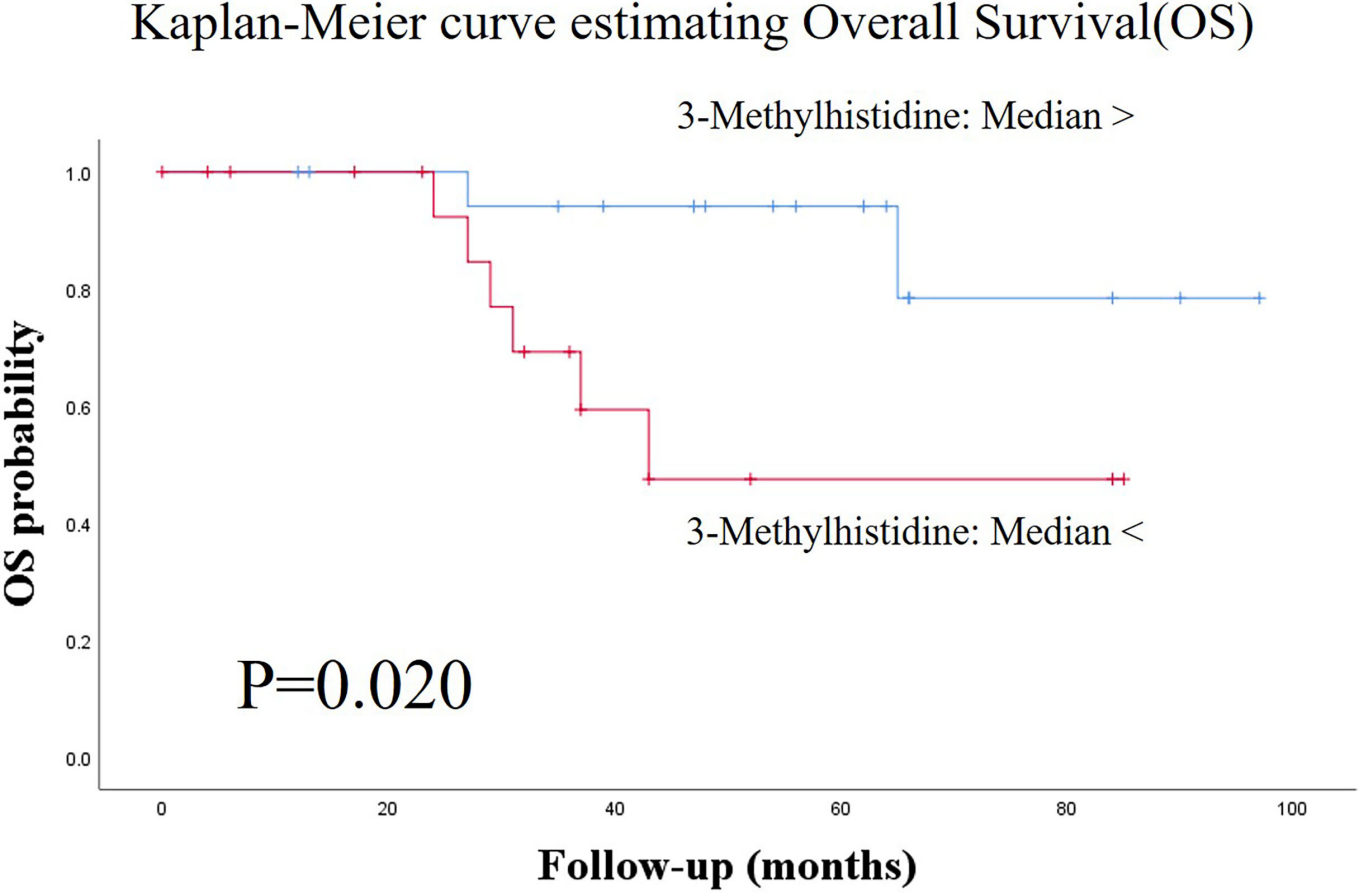
Kaplan-Meier survival curves for estimating overall survival (OS) based on the definitive variable, which is adopted in the Cox hazard model in the validation group. Patients with higher salivary 3-methylhistidine levels (> median) had significantly lower OS rates than those with lower salivary 3-methylhistidine levels (< median) in the test group (p = 0.020). OS, overall survival.

**Figure 3:**
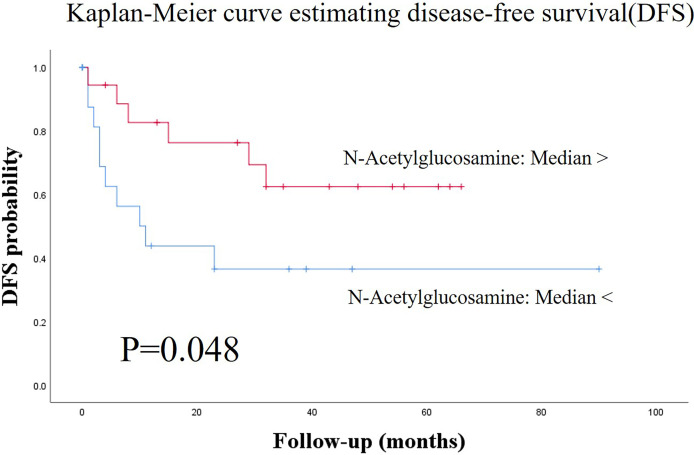
Kaplan-Meier survival curves for estimating disease-free survival (DFS) based on the definitive variable, which is adopted in the Cox hazard model in the validation group. Patients with lower salivary N-acetylglucosamine levels (< median) had significantly lower DFS than those with higher salivary N-acetylglucosamine levels (> median) in the validation group (p = 0.048). DFS, disease-free survival.

**Figure 1** shows the score plots of the PCA of two batches. The distance of the plots is inversely related to the similarities in the metabolite concentration patterns of the two batches. Most of the batch 1 (red) and batch 2 plots (green) converged at similar parts of the score plots. These distributions were indicative of the similarity between samples from batch 1 and batch 2. **Table 1** shows the participants’ characteristics, including age, sex, and smoking habit, as well as clinical parameters, including staging, OSCC antigen levels, standard uptake values of positron emission tomography/computed tomography (CT) in the early/late phases, and follow-up durations. None of the clinical parameters showed significant between-group differences. The median follow-up periods were 57 (range: 3–100) months and 43 (range: 0–97) months in the training and validation groups, respectively. **Supplementary Table 1** shows the unadjusted and adjusted HRs for variables associated with OS in the training group. Univariate analysis of the training data identified proline (HR = 1.001, p = 0.020), carnitine (HR = 1.047, p = 0.042), 5-hydroxylysine (HR = 1.110, p = 0.019), 3-methylhistidine (HR = 3.261, p = 0.035), adenosine (HR = 8.301, p = 0.003), inosine (HR = 1.369, p = 0.040), and *N*-acetylglucosamine (HR = 1.027, p = 0.004) as significant prognostic factors for predicting OS in patients with OSCC. Subsequent multivariate analysis using training data revealed that 3-methylhistidine and 5-hydroxylysine were significant prognostic factors for OS in patients with OSCC (HR = 4.865 and 1.142, p = 0.012 and 0.009, respectively). **Table 2** shows the adjusted HRs for variables associated with OS in the validation group. Two metabolites, 3-methylhistidine and 5-hydroxylysine, were adopted in the multivariate analysis of the validation group, with only 3-methylhistidine being identified as a significant prognostic factor (HR = 1.711, p = 0.048). **Supplementary Table 2** shows the unadjusted and adjusted HRs for variables associated with DFS in the training group. Univariate analysis using training data revealed that creatinine (HR = 1.157, p = 0.048), proline (HR = 1.002, p = 0.029), and *N*-acetylglucosamine (HR = 1.026, p = 0.016) were significant prognostic factors for DFS in OSCC. Subsequent multivariate analysis showed that salivary *N*-acetylglucosamine was a significant prognostic factor for DFS in patients with OSCC (HR = 1.026, p = 0.016). Accordingly, salivary *N*-acetylglucosamine was adopted in the model in the validation group; however, it was not identified as a significant prognostic factor for DFS (HR = 0.988, p = 0.099) (**Table 3**). **Figures 2**, **3** show Kaplan-Meier survival curves for OS and DFS, respectively, based on the definitive variable adopted in the Cox hazard model for the validation group. Participants with higher levels of salivary 3-methylhistidine (> median) had significantly lower OS rates than those with lower levels of salivary 3-methylhistidine (< median) in the validation group (p = 0.020). Participants with lower levels of salivary N-acetylglucosamine (< median) had significantly lower DFS rates than those with higher levels of salivary *N*-acetylglucosamine (> median) in the validation group (p = 0.048). **Supplementary Tables 3, 4** show the correlation coefficient between salivary metabolites and continuous clinical variables in the training and validation groups, respectively. Despite the correlations among age and a few metabolites, most metabolites showed no correlations with stage, early phase standard uptake value, and late phase standard uptake value. **Supplementary Tables 5, 6** show the sex-dependency of salivary metabolites. Only two metabolites (creatinine and indole-3-acetate) showed a significant difference between male and female participants.

**Table 1:** Characteristics of all participants (n = 72).

Variable		Training Group (n = 35)	Validation Group (n = 37)	p-value^†^
Sex	Male (%)	20 (57.1)	18 (48.6)	0.314
	Female (%)	15 (42.9)	19 (51.4)	
Smoking	Yes (%)	2 (5.7)	6 (16.2)	0.149
Stage	0 (CIS) (%)	2 (5.7)	1 (2.7)	0.059
	I (%)	16 (45.7)	8 (21.6)	
	II (%)	6 (17.1)	8 (21.6)	
	III (%)	3 (8.6)	10 (27.0)	
	IV (%)	8 (22.9)	10 (27.0)	
SCC antigen^§^	1.5< (%)	9 (25.7)	8 (21.6)	0.423
	1.5≥ (%)	16 (45.7)	19 (51.3)	
				p-value^‡^
Age	median (min-max)	65.0 (26-89)	69 (23-94)	0.313
Early phase Standard Uptake Value	median (min-max)	10.7 (2.2-23.2)	11.1 (3.0-22.0)	0.245
Late phase Standard Uptake Value	median (min-max)	11.6 (1.8-26.9)	13.44 (4.0-30.0)	0.172
Follow-up period(month)	median (min-max)	55 (3-100)	43 (0-97)	0.101

^†^p-value by chi-square test.

^‡^p-value by Mann-Whitney U-test.

^§^Missing data were 28.6% and 27.0% of each group.

SCC, squamous cell carcinoma.

**Table 2:** Adjusted hazard ratios and 95% confidence intervals for variables associated with overall survival in the validation group.

Variable		Adjusted HR	(95% CI)	p-value
3-Methylhistidine	(per 1 increase)	1.711	1.004-2.916	0.048	*

*statistically significant (p <0.05).

HR, hazard ratio; CI, confidence interval.

Adjusted for variables with p <0.05 in the multivariate analysis in the training group: 3-Methylhistidine and 5-Hydroxylysine.

**Table 3:** Unadjusted hazard ratios and 95% confidence intervals for variables associated with disease-free survival in the validation group.

Variable		Unadjusted HR	(95% CI)	p-value
N-Acetylglucosamine	(per 1 increase)	0.988	0.973-1.002	0.099

HR, hazard ratio; CI, confidence interval.

N-Acetylglucosamine was adopted for the final model in this validation set because only N-acetylglucosamine had a p-value <0.05 in the multivariate analysis in the training group.

## Discussion

This study analyzed the relationships between salivary metabolites and the prognosis of OSCC. We found that salivary 3-methylhistidine was a significant prognostic biomarker for predicting OS in patients with OSCC in both the training and validation groups. OSCC staging, including the TN-stage, and surgical margin status are the most established clinical prognostic factors (19). Imaging-based biomarkers, including CT, magnetic resonance imaging, and F-fluorodeoxyglucose positron emission tomography/CT parameters, are established prognostic factors for OSCC (20, 21). Recently, molecular biomarkers quantified through liquid and tissue biopsy have been reported (2, 22, 23). However, the liquid biopsy applied to blood, rather than saliva, as the biofluid (2, 22, 23). Most surveys were performed using genomics, transcriptomics, or proteomics approaches, rather than a metabolomics approach (2, 24, 25). To our knowledge, this is the first study to identify salivary metabolites for predicting the prognosis of OSCC, which makes our findings significant.

Two studies have used blood metabolomics approaches to identify prognostic biomarkers of OSCC (26, 27). Cadoni et al. reported that 12 serum metabolites, including 3-methylhistidine, were biomarkers for predicting OS in head and neck cancer, including OSCC (27). We found that salivary 3-methylhistidine was a significant prognostic biomarker for OS. Generally, 3-methylhistidine is considered a marker of muscle proteolysis; moreover, increased 3-methylhistidine levels could be biomarkers of frailty and sarcopenia (27, 28). General statuses, including Karnofsky performance status, sarcopenia status, and frailty status, are well-known prognostic factors for OS in head and neck cancer (29–32). Therefore, 3-methylhistidine levels may indicate host factors, such as general status, rather than cancer aggressiveness. Prognostic biomarkers for OSCC, especially tissue-based biomarkers, are based on tumor aggressiveness in general (2, 9, 33, 34). As we mentioned above, our candidate salivary biomarkers, such as 3-methylhistidine, could be derived from the non-cancerous tissue. However, further studies are required to confirm from which tissue our candidate biomarkers are derived. Compared with healthy controls, patients with head and neck cancer have significantly higher serum, but not salivary levels of 3-methylhistidine (35). These reports are consistent with our findings that higher salivary levels of 3-methylhistidine were indicative of poor prognosis of OS in patients with OSCC.

We selected salivary *N*-acetylglucosamine, proline, and creatinine as candidate biomarkers for predicting the prognosis of DFS of OSCC in the training group; however, they were not significant in the validation group. The addition of *N*-acetylglucosamine at the hydroxyl groups of serine and/or threonine residues in cytosolic and nuclear proteins involved in various intracellular processes is involved in cancer cell biology (36–38). However, there have been no reports regarding the prognostic biomarkers of OSCC from this perspective. Proline is considered an indicator of amino acid utilization in tumor tissues (39). Several studies have reported differences in the serum and salivary proline levels between healthy controls and patients with head and neck cancer, including oral cancer (39, 40). These differences in proline levels have been confirmed in renal cell carcinoma and esophageal cancer (39, 41, 42). Although we did not find these salivary metabolites to be significant prognostic biomarkers for predicting DFS in OSCC, future studies are warranted to assess these salivary biomarkers as candidate biomarkers.

A notable strength of this study is its design. After randomly dividing the participants into the training and validation groups, we performed univariate and multivariate analyses to identify prognostic biomarkers in both the groups. In both the groups, the candidate salivary metabolite showed statistical significance. To our knowledge, no studies have performed multivariate analyses to identify prognostic biomarkers of OSCC in the training and validation groups. Despite our small sample size, the aforementioned points can be considered as strengths of this study.

This study has several limitations. First, this study included a small sample size, which could have led to oversight of potentially significant factors or over/underestimation of the results. Second, we combined our data with data derived from two different batches. The use of only one batch to analyze analytes is desirable due to batch effects (43). However, we performed the PCA, which revealed similarities between both batches. Therefore, there were no unexpected batch effects. There is a need for further studies, including multi-center studies, to collect numerous cases all at once. However, it is difficult to collect numerous cases of OSCC simultaneously in Japan given its low prevalence. Third, we did not survey the status of human papillomavirus (HPV). Several types of HPV, including type 16, are related to OSCC, especially its prognosis (44, 45). Patients with OSCC infected with HPV have a better prognosis (44, 45). Jung et al. have revealed that HPV-positive head and neck squamous cell carcinoma cells rely on mitochondrial respiration with decreased glucose metabolism. Contrastingly, smoking-associated/chemically induced HPV-positive head and neck squamous cell carcinoma cells rely heavily on glycolytic pathways (17, 46). Therefore, there could be differences in the profiles of salivary metabolites between HPV-positive and HPV-negative patients with OSCC (17, 46). Further studies are required to collect data regarding HPV infection to determine the prognosis of OSCC.

In conclusion, our assessment of the associations between salivary metabolites and prognosis of OSCC revealed that salivary 3-methylhistidine is a significant biomarker for predicting the prognosis of OS in OSCC.

## Data Availability Statement

The original contributions presented in the study are included in the article/**Supplementary Material**. Further inquiries can be directed to the corresponding author.

## Ethics Statement

The studies involving human participants were reviewed and approved by the Ethics Committee of Yamagata University Faculty of Medicine (#2021-176). Written informed consent for participation was not required for this study in accordance with the national legislation and the institutional requirements.

## Author Contributions

NO, KK, SU, KE, KY, and MI collected the saliva samples. MS conducted metabolomic analysis. SI designed the study. SI, TK, and MS conducted the statistical analysis. SI wrote the main manuscript and prepared all tables and figures. MS, TK, and MI reviewed and edited the manuscript. All authors reviewed the manuscript. All authors contributed to manuscript revision, read, and approved the submitted version.

## Funding

This work was supported by grants from YU-COE(C) from Yamagata University and the Ministry of Education, Culture, Sports, Science and Technology (MEXT) KAKENHI (16K11742, 17K11897, 19K10304, and 20H05743), and research funds from Yamagata Prefectural Government and Tsuruoka City, Japan.

## Conflict of Interest

The authors declare that the research was conducted in the absence of any commercial or financial relationships that could be construed as a potential conflict of interest.

## Publisher’s Note

All claims expressed in this article are solely those of the authors and do not necessarily represent those of their affiliated organizations, or those of the publisher, the editors and the reviewers. Any product that may be evaluated in this article, or claim that may be made by its manufacturer, is not guaranteed or endorsed by the publisher.
