# Supplementary material for: Salivary Metabolomics for Prognosis of Oral Squamous Cell Carcinoma
Source: Front Oncol. 2022 Jan 5;11:789248. doi: 10.3389/fonc.2021.789248 (PMC8769065; doi:10.3389/fonc.2021.789248)
Supplement: Supplementary file 1 [file DataSheet_1.pdf]

Supplementary Tables (1 to 6)

Supplementary Table 1. Adjusted hazard ratios and 95% confidence intervals for variables associated with overall survival in the training group.

Variable		Unadjusted HR	(95% CI)	p-value		Adjusted HR	(95% CI)	p-value
Sex	(female vs. male)	3.015	0.548-16.579	0.204				
Age	(per 1 increase)	1.019	0.964-1.077	0.514				
Stage	(IV, III vs II, I, 0)	1.937	0.390-9.626	0.419				
Smoking habit	(Yes vs No)	0.045	0.000-495062.999	0.708				
SCC antigen	(1.5 > vs <1.5)	2.312	0.318-16.830	0.408				
Early phase Standard Uptake Value	(per 1 increase)	1.086	0.947-1.246	0.239				
Late phase Standard Uptake Value	(per 1 increase)	1.076	0.954-1.214	0.234				
Trimethylamine N-oxide	(per 1 increase)	1.000	0.478-2.095	0.999				
Putrescine (1,4-Butanediamine)	(per 1 increase)	1.007	0.996-1.018	0.236				
GABA	(per 1 increase)	0.785	0.428-1.439	0.434				
Choline	(per 1 increase)	0.996	0.928-1.069	0.914				
Diethanolamine	(per 1 increase)	0.416	0.089-1.936	0.264				
Creatinine	(per 1 increase)	1.004	0.811-1.244	0.971				
Pro	(per 1 increase)	1.001	1.000-1.003	0.020	*			
Betaine	(per 1 increase)	1.068	0.927-1.231	0.363				
5-Aminovalerate	(per 1 increase)	1.000	0.998-1.002	0.828				
Val	(per 1 increase)	0.990	0.954-1.027	0.586				
Nicotinamide	(per 1 increase)	5.122	0.300-87.591	0.259				
Pipecolate	(per 1 increase)	1.101	0.716-1.692	0.662				
N-Acetylputrescine	(per 1 increase)	1.043	0.910-1.197	0.542				
Agmatine	(per 1 increase)	1.568	0.099-24.901	0.750				
Creatine	(per 1 increase)	1.013	0.942-1.088	0.734				
Ile	(per 1 increase)	1.027	0.977-1.079	0.296				
Leu	(per 1 increase)	1.006	0.984-1.029	0.579				
Ornithine	(per 1 increase)	1.001	0.983-1.019	0.933				
Adenine	(per 1 increase)	1.000	0.711-1.406	0.998				
Hypoxanthine	(per 1 increase)	0.952	0.827-1.095	0.487				
Urocanate	(per 1 increase)	0.895	0.638-1.256	0.523				
gamma-Butyrobetaine	(per 1 increase)	1.028	0.893-1.183	0.704				

Spermidine	(per 1 increase)	1.082	0.959-1.221	0.200					
Lys	(per 1 increase)	1.000	0.993-1.006	0.926					
Met	(per 1 increase)	0.979	0.847-1.133	0.778					
Guanine	(per 1 increase)	1.197	0.923-1.553	0.175					
Xanthine	(per 1 increase)	1.037	0.923-1.166	0.536					
His	(per 1 increase)	0.992	0.930-1.058	0.804					
Ala-Ala	(per 1 increase)	1.115	0.827-1.503	0.476					
alpha-Aminoadipate	(per 1 increase)	0.977	0.620-1.540	0.919					
Carnitine	(per 1 increase)	1.047	1.002-1.095	0.042	*				
5-Hydroxylysine	(per 1 increase)	1.110	1.017-1.212	0.019	*	1.142	1.034-1.261	0.009	*
3-Methylguanine	(per 1 increase)	1.197	0.023-62.754	0.929					
7-Methylguanine	(per 1 increase)	0.662	0.004-112.397	0.875					
Phe	(per 1 increase)	0.995	0.960-1.032	0.806					
3-Methylhistidine	(per 1 increase)	3.261	1.089-9.762	0.035	*	4.865	1.415-16.730	0.012	*
Arg	(per 1 increase)	1.009	0.981-1.039	0.527					
Indole-3-acetate	(per 1 increase)	1.019	0.879-1.181	0.804					
Tyr	(per 1 increase)	1.000	0.972-1.029	0.998					
Phosphorylchholine	(per 1 increase)	1.054	0.911-1.220	0.477					
N1-Acetylspermidine	(per 1 increase)	1.475	0.575-3.782	0.419					
N8-Acetylspermidine	(per 1 increase)	11.858	0.157-897.043	0.263					
N-epsilon-Acetyllysine	(per 1 increase)	1.653	0.603-4.532	0.329					
Gly-Leu	(per 1 increase)	0.840	0.365-1.933	0.682					
N6,N6,N6-Trimethyllysine	(per 1 increase)	1.379	0.261-7.286	0.705					
ADMA	(per 1 increase)	1.656	0.160-17.169	0.672					
Spermine	(per 1 increase)	1.164	0.982-1.379	0.081					
o-Acetylcarnitine	(per 1 increase)	2.730	0.941-7.924	0.065					
Trp	(per 1 increase)	1.024	0.796-1.317	0.854					
beta-Ala-Lys	(per 1 increase)	0.837	0.163-4.296	0.831					
N1,N8-Diacetylspermidine	(per 1 increase)	1.920	0.098-37.452	0.667					
Cytidine	(per 1 increase)	0.980	0.309-3.113	0.973					
N1-Acetylspermine	(per 1 increase)	2.556	0.890-7.346	0.081					
Glycerophosphorylcholine	(per 1 increase)	0.920	0.600-1.413	0.705					
Adenosine	(per 1 increase)	8.301	2.034-33.871	0.003	*				
Inosine	(per 1 increase)	1.369	1.015-1.847	0.040	*				

Glu-Glu	(per 1 increase)	1.178	0.736-1.887	0.495	
Guanosine	(per 1 increase)	1.929	0.861-4.326	0.111	
N1,N12-Diacetylspermine	(per 1 increase)	12.442	0.536-288.613	0.116	
Homoserine + Alpha-Methylserine + Thr	(per 1 increase)	1.045	0.505-2.163	0.905	
Taurine	(per 1 increase)	0.999	0.993-1.006	0.795	
Hydroxyproline	(per 1 increase)	0.940	0.482-1.832	0.855	
Trigonelline	(per 1 increase)	0.858	0.434-1.694	0.658	
Ethanolamine phosphate	(per 1 increase)	0.979	0.941-1.019	0.300	
Proline betaine	(per 1 increase)	1.327	0.704-2.499	0.381	
Gln	(per 1 increase)	1.005	0.990-1.020	0.535	
O-Acetylserine + Glu	(per 1 increase)	1.010	0.986-1.035	0.416	
Citrulline	(per 1 increase)	0.996	0.967-1.025	0.770	
N-Acetylglucosamine	(per 1 increase)	1.027	1.008-1.046	0.004	*

*statistically significant (p <0.05)

HR, hazard ratio; CI, confidence interval; SCC: squamous cell carcinoma

Adjusted for variables with a p-value <0.05 in the univariate analysis

Supplementary Table 2. Adjusted hazard ratios and 95% confidence intervals for variables associated with disease-free survival in the training group.

Variable		Unadjusted HR	(95% CI)	p-value		Adjusted HR	(95% CI)	p-value
Sex	(female vs male)	0.682	0.132-3.532	0.648				
Age	(per 1 increase)	0.982	0.941-1.025	0.410				
Stage	(IV, III vs II, I, 0)	1.62	0.361-7.266	0.529				
Smoking	(Yes vs No)	3.037	0.353-26.157	0.312				
SCC antigen	(1.5 > vs <1.5)	1.981	0.399-9.825	0.403				
Early phase Standard Uptake Value	(per 1 increase)	1.097	0.965-1.248	0.157				
Late phase Standard Uptake Value	(per 1 increase)	1.100	0.976-1.239	0.118				
Trimethylamine N-oxide	(per 1 increase)	1.099	0.596-2.027	0.762				
Putrescine(1,4-Butanediamine)	(per 1 increase)	1.008	0.997-1.018	0.162				
GABA	(per 1 increase)	0.852	0.505-1.438	0.548				
Choline	(per 1 increase)	1.012	0.957-1.070	0.677				
Diethanolamine	(per 1 increase)	1.058	0.438-2.552	0.901				
Creatinine	(per 1 increase)	1.157	1.001-1.338	0.048	*			
Pro	(per 1 increase)	1.002	1.000-1.003	0.029	*			
Betaine	(per 1 increase)	1.015	0.885-1.164	0.835				
5-Aminovalerate	(per 1 increase)	1.000	0.998-1.002	0.984				
Val	(per 1 increase)	0.992	0.964-1.021	0.574				
Nicotinamide	(per 1 increase)	0.019	0.000-17.405	0.255				
Pipecolate	(per 1 increase)	1.091	0.715-1.666	0.686				
N-Acetylputrescine	(per 1 increase)	1.035	0.932-1.149	0.522				
Agmatine	(per 1 increase)	1.180	0.122-11.371	0.886				
Creatine	(per 1 increase)	0.945	0.848-1.054	0.310				
Ile	(per 1 increase)	1.022	0.978-1.068	0.328				
Leu	(per 1 increase)	1.007	0.987-1.028	0.486				
Ornithine	(per 1 increase)	1.002	0.985-1.019	0.815				
Adenine	(per 1 increase)	0.885	0.512-1.530	0.661				
Hypoxanthine	(per 1 increase)	1.000	0.925-1.082	0.991				
Urocanate	(per 1 increase)	1.015	0.931-1.107	0.735				
gamma-Butyrobetaine	(per 1 increase)	1.033	0.914-1.168	0.605				

Spermidine	(per 1 increase)	0.921	0.750-1.131	0.431
Lys	(per 1 increase)	1.000	0.996-1.004	0.874
Met	(per 1 increase)	0.989	0.873-1.120	0.860
Guanine	(per 1 increase)	1.088	0.820-1.445	0.558
Xanthine	(per 1 increase)	1.017	0.926-1.118	0.724
His	(per 1 increase)	1.010	0.960-1.062	0.706
Ala-Ala	(per 1 increase)	1.045	0.807-1.353	0.741
alpha-Aminoadipate	(per 1 increase)	0.911	0.574-1.445	0.691
Carnitine	(per 1 increase)	1.020	0.978-1.064	0.365
5-Hydroxylysine	(per 1 increase)	1.046	0.965-1.134	0.273
3-Methylguanine	(per 1 increase)	2.506	0.151-41.503	0.521
7-Methylguanine	(per 1 increase)	0.692	0.005-88.494	0.882
Phe	(per 1 increase)	1.004	0.977-1.031	0.795
3-Methylhistidine	(per 1 increase)	1.760	0.523-5.921	0.361
Arg	(per 1 increase)	0.993	0.960-1.027	0.686
Indole-3-acetate	(per 1 increase)	1.041	0.934-1.161	0.467
Tyr	(per 1 increase)	1.001	0.979-1.023	0.949
Phosphorylchholine	(per 1 increase)	0.976	0.830-1.147	0.764
N1-Acetylspermidine	(per 1 increase)	0.382	0.039-3.725	0.407
N8-Acetylspermidine	(per 1 increase)	4.409	0.150-129.654	0.390
N-epsilon-Acetyllysine	(per 1 increase)	1.394	0.577-3.368	0.460
Gly-Leu	(per 1 increase)	0.987	0.580-1.678	0.961
N6,N6,N6-Trimethyllysine	(per 1 increase)	0.912	0.177-4.703	0.912
ADMA	(per 1 increase)	1.951	0.450-8.451	0.372
Spermine	(per 1 increase)	1.065	0.902-1.257	0.459
o-Acetylcarnitine	(per 1 increase)	1.135	0.388-3.317	0.817
Trp	(per 1 increase)	1.112	0.925-1.336	0.259
beta-Ala-Lys	(per 1 increase)	0.984	0.285-3.403	0.980
N1,N8-Diacetylspermidine	(per 1 increase)	0.638	0.022-18.130	0.793
Cytidine	(per 1 increase)	0.825	0.261-2.606	0.743
N1-Acetylspermine	(per 1 increase)	1.875	0.688-5.109	0.219
Glycerophosphorylcholine	(per 1 increase)	0.621	0.192-2.012	0.427
Adenosine	(per 1 increase)	3.738	0.783-17.839	0.098
Inosine	(per 1 increase)	1.133	0.830-1.548	0.432
Glu-Glu	(per 1 increase)	1.030	0.635-1.671	0.903

Guanosine	(per 1 increase)	1.132	0.452-2.836	0.791					
N1,N12-Diacetylspermine	(per 1 increase)	0.127	0.000-106.548	0.548					
Homoserine + Alpha-Methylserine + Thr	(per 1 increase)	0.906	0.452-1.814	0.780					
Taurine	(per 1 increase)	1.000	0.994-1.005	0.895					
Hydroxyproline	(per 1 increase)	0.825	0.436-1.563	0.555					
Trigonelline	(per 1 increase)	0.833	0.389-1.785	0.638					
Ethanolamine phosphate	(per 1 increase)	0.991	0.960-1.023	0.563					
Proline betaine	(per 1 increase)	1.234	0.677-2.251	0.492					
Gln	(per 1 increase)	1.005	0.993-1.017	0.404					
O-Acetylserine + Glu	(per 1 increase)	1.002	0.979-1.025	0.889					
Citrulline	(per 1 increase)	1.009	0.992-1.027	0.314					
N-Acetylglucosamine	(per 1 increase)	1.026	1.005-1.048	0.016	*	1.026	1.005-1.048	0.016	*

*statistically significant (p <0.05)

HR, hazard ratio; CI, confidence interval; SCC: squamous cell carcinoma;

Adjusted for the variables with p <0.05 in the univariate analysis

Supplementary Table 3. Correlation coefficient between salivary metabolites and clinical variables (continuous variables) in the training group.

	Age		Stage (0 to IV)		Early Phase Standard Uptake Value	Late Phase Standard Uptake Value	
Trimethylamine N-oxide	0.394	*	-0.009		0.072	0.046	
Putrescine(1,4-Butanediamine)	0.450	**	-0.016		0.180	0.262	
GABA	-0.111		0.101		-0.039	-0.040	
Choline	0.295		0.160		0.000	0.063	
Diethanolamine	-0.044		-0.363	*	-0.191	-0.232	
Creatinine	0.194		0.369	*	0.229	0.330	
Pro	0.292		-0.187		0.079	0.133	
Betaine	0.211		0.207		0.035	0.098	
5-Aminovalerate	0.290		0.081		0.338	0.369	*
Val	-0.301		0.155		-0.113	-0.194	
Nicotinamide	0.021		0.008		-0.160	-0.220	
Pipecolate	-0.059		0.284		0.041	0.006	
N-Acetylputrescine	0.357	*	0.041		0.082	0.156	
Agmatine	0.004		-0.383	*	-0.132	-0.100	
Creatine	0.443	**	0.197		0.033	0.103	
Ile	0.333		0.109		0.180	0.244	
Leu	0.341	*	0.123		0.155	0.210	
Ornithine	0.431	*	-0.067		0.137	0.162	
Adenine	0.466	**	0.261		0.104	0.158	
Hypoxanthine	0.122		0.229		0.010	0.003	
Urocanate	0.269		-0.023		0.056	0.099	
gamma-Butyrobetaine	0.397	*	0.036		0.135	0.180	
Spermidine	0.259		0.074		-0.111	-0.100	
Lys	0.303		-0.043		0.026	0.121	
Met	0.310		0.208		0.133	0.172	
Guanine	0.100		0.245		0.278	0.363	
Xanthine	0.141		0.139		-0.085	-0.023	
His	0.227		0.040		-0.044	-0.017	
Ala-Ala	0.258		-0.028		-0.062	0.037	
alpha-Aminoadipate	0.018		-0.041		-0.214	-0.244	
Carnitine	0.264		0.129		0.175	0.247	
5-Hydroxylysine	0.166		0.140		0.241	0.316	

3-Methylguanine	0.380		-0.037	0.134	0.210
7-Methylguanine	0.167		0.316	0.121	0.183
Phe	0.280		-0.051	-0.017	0.021
3-Methylhistidine	0.424	*	0.033	0.111	0.142
Arg	0.297		-0.029	0.212	0.223
Indole-3-acetate	0.245		-0.044	0.181	0.210
Tyr	0.337	*	-0.059	0.068	0.096
Phosphorylchholine	0.333		0.025	0.228	0.286
N1-Acetylspermidine	0.337	*	-0.027	0.131	0.170
N8-Acetylspermidine	0.177		-0.123	-0.068	-0.007
N-epsilon-Acetyllysine	0.106		-0.071	-0.197	-0.060
Gly-Leu	0.187		0.016	-0.057	-0.052
N6,N6,N6-Trimethyllysine	0.286		-0.044	0.031	0.134
ADMA	0.061		0.112	0.058	0.060
Spermine	0.374	*	0.185	0.201	0.233
o-Acetylcarnitine	0.116		0.294	0.143	0.198
Trp	0.300		0.150	0.082	0.117
beta-Ala-Lys	0.242		0.017	-0.111	-0.098
N1,N8-Diacetylspermidine	0.394	*	-0.167	0.039	0.103
Cytidine	0.344	*	-0.165	-0.279	-0.272
N1-Acetylspermine	0.367	*	0.079	0.163	0.185
Glycerophosphorylcholine	0.342	*	0.076	0.046	0.047
Adenosine	0.424	*	0.044	0.096	0.190
Inosine	0.230		0.136	0.114	0.179
Glu-Glu	0.233		-0.066	-0.001	-0.003
Guanosine	0.332		0.218	0.169	0.284
N1,N12-Diacetylspermine	0.435	**	-0.125	0.060	0.098
Homoserine + Alpha-Methylserine + Thr	0.310		0.337	0.034	0.101
Taurine	0.141		0.194	-0.098	-0.077
Hydroxyproline	0.356	*	-0.029	-0.120	-0.124
Trigonelline	0.033		0.021	0.202	0.237
Ethanolamine phosphate	0.090		0.020	0.067	0.027
Proline betaine	0.152		-0.220	-0.053	0.001
Gln	0.268		0.029	0.045	0.093

O-Acetylserine + Glu	0.320	0.229	0.049	0.137
Citrulline	0.194	0.077	0.026	0.128
N-Acetylglucosamine	0.300	-0.202	0.008	0.029

*statistically significant ($p < 0.05$)
**statistically significant ($p < 0.01$)

SCC: squamous cell carcinoma

Supplementary Table 4. Correlation coefficient between salivary metabolites and clinical variables (continuous variables) in the validation group.

	Age		Stage (0 to IV)		Early Phase Standard Uptake Value	Late Phase Standard Uptake Value	
Trimethylamine N-oxide	0.165		0.053		-0.099	0.013	
Putrescine (1,4-Butanediamine)	0.349	*	0.324		0.136	0.177	
GABA	0.066		0.21		-0.024	0.099	
Choline	0.261		0.273		0.026	0.137	
Diethanolamine	0.032		-0.186		-0.291	-0.398	*
Creatinine	0.145		0.135		-0.079	0.007	
Pro	0.402	*	0.042		-0.017	0.003	
Betaine	0.244		-0.008		-0.061	0.033	
5-Aminovalerate	0.186		0.093		-0.066	-0.01	
Val	0.036		0.286		-0.027	0.068	
Nicotinamide	0.023		-0.167		-0.075	-0.047	
Pipecolate	0.195		0.157		-0.057	0.049	
N-Acetylputrescine	0.193		0.24		0.017	0.073	
Agmatine	0.125		0.144		-0.103	-0.175	
Creatine	0.348	*	0.003		-0.038	0.074	
Ile	0.091		0.215		-0.092	0.007	
Leu	0.168		0.263		-0.048	0.059	
Ornithine	0.29		0.292		-0.164	-0.092	
Adenine	0.174		0.187		0.25	0.285	
Hypoxanthine	0.045		0.361	*	0.001	0.106	
Urocanate	0.263		0.159		-0.208	-0.136	
gamma-Butyrobetaine	0.247		0.286		0.11	0.145	
Spermidine	0.244		0.345	*	0.05	0.122	
Lys	0.194		0.114		-0.164	-0.109	
Met	-0.027		0.316		-0.035	0.052	
Guanine	0.072		0.079		0.24	0.365	*
Xanthine	0.07		0.092		-0.078	-0.137	
His	0.285		0.217		-0.075	0.012	
Ala-Ala	0.212		0.109		0.001	0.094	
alpha-Aminoadipate	0.091		0.257		0.036	0.15	
Carnitine	0.252		-0.052		0.164	0.166	
5-Hydroxylysine	0.094		-0.179		0.114	0.073	

3-Methylguanine	0.119		0.163		-0.409		-0.445	*
7-Methylguanine	0.171		0.056		-0.14		-0.07	
Phe	0.196		0.168		-0.131		-0.036	
3-Methylhistidine	0.279		0.326	*	0.275		0.399	*
Arg	0.167		-0.18		-0.046		-0.128	
Indole-3-acetate	0.172		0.204		0.147		0.099	
Tyr	0.282		0.175		-0.073		0.019	
Phosphorylchholine	0.133		-0.198		-0.32		-0.235	
N1-Acetylspermidine	0.231		0.222		0.158		0.139	
N8-Acetylspermidine	0.177		0.193		-0.132		-0.027	
N-epsilon-Acetyllysine	0.247		0.091		-0.067		0.012	
Gly-Leu	-0.052		0.188		-0.087		0.024	
N6,N6,N6-Trimethyllysine	0.185		0.226		0.145		0.222	
ADMA	0.351	*	0.171		0.423	*	0.43	*
Spermine	0.286		0.111		0.232		0.346	
o-Acetylcarnitine	0.392	*	0.039		0.289		0.401	*
Trp	0.152		0.314		-0.015		0.098	
beta-Ala-Lys	0.077		0.047		-0.157		-0.172	
N1,N8-Diacetylspermidine	0.193		0.008		-0.082		-0.117	
Cytidine	-0.008		0.194		-0.277		-0.271	
N1-Acetylspermine	0.079		0.098		0.031		0.117	
Glycerophosphorylcholine	0.246		-0.037		0.11		0.265	
Adenosine	0.234		0.036		0.214		0.188	
Inosine	0.103		0.11		0.155		0.205	
Glu-Glu	-0.016		0.008		-0.095		-0.067	
Guanosine	0.025		0.225		0.107		0.168	
N1,N12-Diacetylspermine	0.155		0.154		-0.016		-0.044	
Homoserine + Alpha-Methylserine + Thr	0.149		0.471	*	0.136		0.231	
Taurine	0.124		0.323		0.171		0.31	
Hydroxyproline	0.189		0.057		-0.007		0.117	
Trigonelline	-0.036		-0.146		-0.023		0.059	
Ethanolamine phosphate	0.11		0.123		0.178		0.322	
Proline betaine	0.074		-0.353	*	-0.133		-0.16	
Gln	0.218		0.165		-0.078		0.008	
O-Acetylserine + Glu	0		0.254		-0.041		0.001	

Citrulline	0.2	0.228	-0.054	-0.008
N-Acetylglucosamine	0.033	-0.133	-0.01	-0.125

*statistically significant ($p < 0.05$)

SCC: squamous cell carcinoma;

Supplementary Table 5. Comparison of salivary metabolites between sex in the training group.

	Male						Female						p-value	
Trimethylamine N-oxide	0.192077069	(	0.16109659	-	0.360853213	)	0.154265021	(	0	-	0.360052612	)	0.762	
Putrescine (1,4-Butanediamine)	62.0286545	(	34.94451331	-	98.17473535	)	61.3069384	(	44.13986697	-	159.989776	)	0.790	
GABA	0	(	0	-	0	)	0	(	0	-	0	)	0.834	
Choline	10.78563204	(	8.584367175	-	18.82644403	)	10.84600977	(	6.875571234	-	14.58641934	)	0.739	
Diethanolamine	0	(	0	-	1.519442313	)	1.268333857	(	0.883305781	-	1.521147909	)	0.471	
Creatinine	5.379958024	(	4.65355555	-	9.257319862	)	3.532461213	(	2.313505785	-	5.023849867	)	0.006	*
Pro	49.16881456	(	18.56553302	-	119.2367685	)	56.23927392	(	45.83498451	-	528.4654558	)	0.714	
Betaine	6.519774896	(	5.250823991	-	10.30193033	)	5.697240605	(	4.551399552	-	8.689552817	)	0.182	
5-Aminovalerate	78.73095041	(	48.8736377	-	165.9551304	)	147.8854653	(	48.71331043	-	288.8755126	)	0.841	
Val	0	(	0	-	2.297159176	)	0	(	0	-	0	)	0.774	
Nicotinamide	0	(	0	-	0.268432119	)	0.174915862	(	0	-	0.385999565	)	0.551	
Pipecolate	0	(	0	-	0.550567073	)	0	(	0	-	0	)	0.693	
N-Acetylputrescine	3.1814359	(	1.552308902	-	7.007144165	)	2.474883934	(	1.460886101	-	6.825007088	)	0.594	
Agmatine	0	(	0	-	0.373372433	)	0.18155707	(	0	-	0.454592677	)	0.135	
Creatine	17.64577397	(	12.699649	-	22.53347237	)	18.69125286	(	14.37448061	-	20.58670035	)	0.947	
Ile	5.598395961	(	2.107350983	-	13.68212397	)	7.866219398	(	2.421746873	-	12.06275188	)	1.000	
Leu	10.49816066	(	5.460912374	-	26.01116519	)	12.61928335	(	5.846220501	-	20.17942543	)	0.641	
Ornithine	17.75384049	(	11.26486375	-	38.96617658	)	19.15770377	(	15.08404072	-	67.24294415	)	0.947	
Adenine	1.176279536	(	0.929926087	-	2.606516837	)	1.369831494	(	0.887336043	-	2.300096682	)	0.973	
Hypoxanthine	1.341156878	(	0.536475958	-	2.358454293	)	0.899501981	(	0.598164194	-	1.274096906	)	0.947	
Urocanate	3.852186485	(	2.149885745	-	5.005666826	)	3.896133682	(	1.873289003	-	5.786951395	)	0.907	
gamma-Butyrobetaine	3.050031932	(	1.601319934	-	6.872510776	)	2.573067352	(	1.593407454	-	4.478339893	)	0.617	
Spermidine	3.237391537	(	2.265278261	-	7.609441117	)	2.397028786	(	0.75933578	-	6.646078938	)	0.617	
Lys	98.48407848	(	21.85606335	-	136.6606026	)	108.7712996	(	24.57307958	-	177.1520162	)	1.000	
Met	1.653002965	(	0.832418771	-	2.653605014	)	1.288366992	(	0.803147948	-	1.60090862	)	0.203	
Guanine	1.236003373	(	0.7951638	-	2.142912887	)	0.871550649	(	0.562375714	-	2.359055506	)	0.594	
Xanthine	4.326775317	(	2.776016453	-	6.935183194	)	2.908684505	(	0	-	5.631751325	)	0.282	
His	9.512318742	(	4.880676456	-	12.69533008	)	8.950594084	(	6.597868489	-	16.47639544	)	0.790	
Ala-Ala	1.70175451	(	1.021360117	-	2.471736759	)	1.549336962	(	0	-	1.870865688	)	0.481	
alpha-Aminoadipate	0	(	0	-	0	)	0	(	0	-	0	)	0.875	
Carnitine	11.87786692	(	10.31495043	-	31.94107419	)	14.4233538	(	10.46891691	-	26.75263337	)	0.527	
5-Hydroxylysine	6.291961458	(	5.033048345	-	12.71909208	)	7.747130336	(	5.249189651	-	13.56437757	)	0.946	

3-Methylguanine	0	(0 - 0.161510615)	0.081963521	(0 - 0.291295503)	0.482
7-Methylguanine	0	(0 - 0.18149521)	0.098844839	(0 - 0.172033386)	0.415
Phe	22.15665468	(7.777592319 - 30.36276146)	19.87415664	(8.439212682 - 35.6811574)	0.894
3-Methylhistidine	0	(0 - 0.579294309)	0	(0 - 0.275288576)	0.971
Arg	27.89313031	(12.28635081 - 36.27299036)	27.79712781	(20.82122706 - 48.87556309)	0.617
Indole-3-acetate	3.545735482	(0.656724818 - 8.199000907)	3.842535798	(2.397450106 - 10.13173673)	0.973
Tyr	30.79892745	(11.25285714 - 38.80660577)	26.41411028	(14.15984922 - 44.79197242)	0.764
Phosphorylchholine	13.28175486	(8.388932464 - 15.96101432)	12.49586409	(8.225578428 - 15.01680554)	0.698
N1-Acetylspermidine	0.262062469	(0.12095406 - 0.46391373)	0.163525645	(0.132631606 - 0.326137182)	0.776
N8-Acetylspermidine	0.072131773	(0 - 0.140773525)	0.068163148	(0 - 0.149836772)	0.946
N-epsilon-Acetyllysine	0.566085984	(0.295805099 - 1.122460148)	0.404782765	(0.208889118 - 0.662599621)	0.513
Gly-Leu	0	(0 - 0.08155391)	0	(0 - 0)	0.910
N6,N6,N6-Trimethyllysine	0.243728602	(0 - 0.614328037)	0.219644481	(0 - 0.524421441)	0.627
ADMA	0	(0 - 0.252628092)	0	(0 - 0)	0.544
Spermine	1.919634154	(0.723706344 - 4.528328968)	1.82831178	(0.983697228 - 2.706912181)	0.689
o-Acetylcarnitine	0.738961881	(0.441352348 - 0.971662379)	0.596293825	(0.37656781 - 1.140112663)	0.973
Trp	2.590253575	(1.403333082 - 4.153821312)	1.622001052	(1.206860007 - 1.986352147)	0.117
beta-Ala-Lys	0	(0 - 0.535752882)	0.183128378	(0 - 0.442752665)	0.773
N1,N8-Diacetylspermidine	0.177493963	(0.119297256 - 0.224910789)	0.126164423	(0.059599546 - 0.216010063)	0.867
Cytidine	0	(0 - 0.258225077)	0.317449635	(0 - 0.678441053)	0.306
N1-Acetylspermine	0.143474508	(0.087766394 - 0.613214086)	0.124798712	(0 - 0.244441048)	0.108
Glycerophosphorylcholine	0.47197221	(0.272119074 - 1.596836584)	0.621124065	(0.490544291 - 1.318309018)	0.480
Adenosine	0.200325247	(0.119723709 - 0.724439876)	0.23520736	(0.126007895 - 0.516400048)	0.763
Inosine	1.994529732	(1.402356094 - 4.372984167)	1.098239575	(0 - 1.886518114)	0.161
Glu-Glu	0.375661818	(0 - 0.582239188)	0.448880532	(0 - 0.993782433)	0.943
Guanosine	0.737736644	(0 - 1.217726649)	0.68670727	(0 - 0.881779959)	0.652
N1,N12-Diacetylspermine	0.089884829	(0.061402228 - 0.192713977)	0.065448572	(0.043092666 - 0.128963992)	0.854
Homoserine + Alpha-Methylserine + Thr	1.643718949	(1.13177777 - 2.644983259)	1.628491314	(1.109394079 - 1.680933537)	0.739
Taurine	46.23739592	(33.53899114 - 98.04018173)	28.5697632	(0 - 48.20180505)	0.423
Hydroxyproline	0	(0 - 1.856984868)	0	(0 - 0.881425526)	0.585
Trigonelline	0.414022862	(0.104250182 - 0.641681056)	0.238258621	(0 - 0.606174738)	0.547
Ethanolamine phosphate	22.09803455	(10.47589861 - 37.93173724)	13.14678784	(2.81809896 - 28.57117074)	0.629
Proline betaine	0.13571639	(0.110578325 - 0.425845212)	0.242405609	(0.129482937 - 0.401836997)	0.763
Gln	36.23324727	(22.52477024 - 66.05118413)	40.24550638	(27.72744614 - 89.10500869)	0.764

O-Acetylserine + Glu	28.97191429	(	19.37023833	-	47.29025336	)	19.22928092	(	17.59595177	-	35.65857936	)	0.405
Citrulline	31.43292992	(	10.08957085	-	37.92797402	)	13.19316914	(	7.792833455	-	40.55624273	)	0.334
N-Acetylglucosamine	38.73793526	(	23.767835	-	64.33656452	)	49.26747021	(	34.38163375	-	63.58731041	)	0.960

*statistically significant (p <0.05)

SCC: squamous cell carcinoma;

Supplementary Table 6. Comparison of salivary metabolites between sex in the validation group.

	Male	Female	p-value
Trimethylamine N-oxide	0.544998763 (0.185102655 - 0.851210841)	0.206636587 (0.114001037 - 0.314082123)	0.259
Putrescine (1,4-Butanediamine)	54.36047087 (39.63338776 - 109.8494919)	101.2258694 (30.63433467 - 196.0037562)	0.514
GABA	0 (0 - 0)	0 (0 - 0)	0.682
Choline	8.5903498 (5.586488227 - 12.85005498)	7.230404721 (5.346986992 - 28.92311379)	0.543
Diethanolamine	0.495202372 (0 - 1.249636242)	0 (0 - 1.097371992)	0.925
Creatinine	6.335580414 (4.835014821 - 7.401412193)	3.534571739 (1.897508511 - 5.355605377)	0.013 *
Pro	121.9077551 (47.64966176 - 264.1080812)	70.8910572 (26.34254389 - 188.1357876)	0.627
Betaine	6.342190019 (4.502028465 - 6.663333695)	6.02475257 (3.818207234 - 8.436331158)	0.952
5-Aminovalerate	97.75524531 (34.9899719 - 223.6315433)	102.6373188 (37.23164396 - 147.9839576)	0.855
Val	0 (0 - 0)	0 (0 - 143.9296032)	0.379
Nicotinamide	0.351193555 (0.249073961 - 0.475591775)	0.197116051 (0 - 0.297917834)	0.583
Pipecolate	0.243207546 (0 - 0.578961491)	0.484696403 (0 - 1.093280945)	0.641
N-Acetylputrescine	2.972918026 (0.948582545 - 7.573200654)	3.376140413 (1.213020432 - 9.504976623)	0.903
Agmatine	0 (0 - 0.383748012)	0 (0 - 0.277695193)	0.329
Creatine	16.47302397 (9.125434224 - 17.09871525)	10.08278838 (8.863985021 - 25.74978901)	0.485
Ile	3.158053813 (1.816408905 - 6.840915745)	3.351996804 (1.15726282 - 9.278417578)	0.927
Leu	5.346508351 (4.038931617 - 15.87532214)	6.523419351 (3.674638002 - 25.57520453)	0.738
Ornithine	17.40458494 (11.94085721 - 29.04436908)	16.34778022 (11.7149628 - 26.67309902)	0.915
Adenine	1.243166107 (1.064023862 - 1.38183071)	1.162103292 (0.980340937 - 1.519398674)	0.274
Hypoxanthine	0.810402468 (0.42231993 - 1.850117171)	0.711873265 (0.506082681 - 2.339014861)	0.952
Urocanate	2.968605328 (1.745164406 - 7.200225066)	2.270888389 (1.156272798 - 4.897817981)	0.274
gamma-Butyrobetaine	2.829348768 (0.865208343 - 3.432446455)	3.930342709 (1.711710442 - 8.747067004)	0.429
Spermidine	2.176849224 (1.499777137 - 2.824650209)	3.65115684 (1.631939674 - 8.652938539)	0.191
Lys	69.68797414 (56.86788412 - 121.0132879)	69.07057521 (17.62765499 - 213.1234265)	0.738
Met	0.556956687 (0 - 2.394944214)	0 (0 - 4.140415724)	0.815
Guanine	1.483447956 (1.061601346 - 1.820345758)	1.722264759 (0.757618766 - 2.24229591)	0.523
Xanthine	3.045851336 (0 - 4.639215802)	2.454068964 (1.918336691 - 13.41882796)	0.073
His	10.36588747 (7.251988844 - 13.06174972)	6.183279881 (4.170501278 - 12.4312845)	0.761
Ala-Ala	1.637931244 (1.400742851 - 1.923138719)	1.283944087 (1.125414771 - 4.232000983)	0.903
alpha-Aminoadipate	0 (0 - 0)	0 (0 - 0)	0.371
Carnitine	11.12488899 (8.401354198 - 21.65978324)	11.72846348 (7.729288489 - 23.8279287)	0.316
5-Hydroxylysine	6.350545769 (5.423949219 - 11.00856168)	5.996649187 (4.52337326 - 9.540652412)	0.683

3-Methylguanine	0.083778105	(	0	-	0.197854236	)	0	(	0	-	0.526702305	)	0.664	
7-Methylguanine	0	(	0	-	0	)	0.094531466	(	0	-	0.204898743	)	0.498	
Phe	15.25960381	(	9.513081824	-	24.59593462	)	15.79529577	(	8.842891799	-	24.27000329	)	0.927	
3-Methylhistidine	0	(	0	-	0	)	0.561129478	(	0	-	0.993097144	)	0.144	
Arg	19.15380287	(	14.68527404	-	35.98989988	)	10.0654595	(	9.219421471	-	22.97089892	)	0.543	
Indole-3-acetate	2.55431475	(	1.467635235	-	3.937708936	)	4.650847532	(	2.87407809	-	17.50461896	)	0.039	*
Tyr	24.9819293	(	12.37705242	-	36.24187154	)	16.64399821	(	11.85757762	-	45.65971323	)	0.976	
Phosphorylchholine	13.35563661	(	5.662477982	-	19.25009229	)	9.315402039	(	4.160695887	-	15.63790719	)	0.495	
N1-Acetylspermidine	0.130911881	(	0.080403752	-	0.168190438	)	0.293148637	(	0.093370951	-	0.613468208	)	0.083	
N8-Acetylspermidine	0	(	0	-	0.086666771	)	0.083279616	(	0	-	0.126820476	)	0.555	
N-epsilon-Acetyllysine	0.394464037	(	0	-	0.754538785	)	0.437851258	(	0.317856097	-	1.563257624	)	0.203	
Gly-Leu	0	(	0	-	0	)	0	(	0	-	0.335673517	)	0.934	
N6,N6,N6-Trimethyllysine	0.178131955	(	0	-	0.324633102	)	0.190121851	(	0.138026488	-	1.390810139	)	0.413	
ADMA	0	(	0	-	0	)	0	(	0	-	0.109901727	)	0.437	
Spermine	1.806553563	(	0.385131692	-	2.369229138	)	1.971980164	(	1.076454086	-	3.632829695	)	0.100	
o-Acetylcarnitine	0.497182817	(	0.385312871	-	1.02999524	)	0.420636355	(	0.314699577	-	0.794536614	)	0.784	
Trp	1.489456612	(	1.017979237	-	2.652226681	)	1.525459827	(	0.885932571	-	4.101388047	)	0.738	
beta-Ala-Lys	0	(	0	-	0.608422222	)	0	(	0	-	0.514236128	)	0.809	
N1,N8-Diacetylspermidine	0.158134451	(	0.056985638	-	0.292275064	)	0.143903197	(	0.076645813	-	0.314877407	)	0.670	
Cytidine	0.734806432	(	0	-	1.056288047	)	0	(	0	-	0.376560011	)	0.058	
N1-Acetylspermine	0.116099779	(	0	-	0.227677875	)	0.112507358	(	0.054661121	-	0.531166571	)	0.197	
Glycerophosphorylcholine	0.511875813	(	0.151641491	-	4.214612464	)	0.956304387	(	0.720396607	-	1.553663159	)	0.255	
Adenosine	0.196635601	(	0.086446188	-	0.398729931	)	0.268538585	(	0.246905656	-	0.377019091	)	0.626	
Inosine	1.67014276	(	1.54953903	-	2.856193708	)	1.975577734	(	1.419600638	-	3.957922225	)	0.551	
Glu-Glu	0.40981294	(	0	-	0.513990376	)	0.511487636	(	0	-	0.747875163	)	0.710	
Guanosine	0.858131341	(	0	-	1.772200021	)	0.589717798	(	0	-	0.809680119	)	0.599	
N1,N12-Diacetylspermine	0.074505812	(	0.035767227	-	0.125120207	)	0.050015376	(	0.03395387	-	0.202624822	)	0.670	
Homoserine + Alpha-Methylserine + Thr	1.456146644	(	0.872019386	-	2.280427808	)	2.110483601	(	0.939166295	-	3.667858545	)	0.352	
Taurine	22.24480912	(	0	-	57.76115951	)	44.15659123	(	18.36960125	-	141.9195601	)	0.345	
Hydroxyproline	0	(	0	-	2.716718787	)	0	(	0	-	2.284582612	)	0.923	
Trigonelline	0.134882628	(	0	-	0.706622139	)	0.089756343	(	0	-	0.376540078	)	1.000	
Ethanolamine phosphate	30.14533149	(	12.45946657	-	33.8681926	)	13.64769792	(	7.376015796	-	31.18749689	)	0.503	
Proline betaine	0.21250022	(	0.052598084	-	0.47940169	)	0.166438692	(	0.050427328	-	0.43113523	)	0.683	
Gln	41.42218476	(	25.13422665	-	49.3597066	)	31.04950939	(	26.09585216	-	100.9130455	)	0.976	

O-Acetylserine + Glu	25.03948777	(	20.47446926	-	27.77689909	)	20.16790128	(	16.3343599	-	51.83504608	)	0.577
Citrulline	13.74908196	(	8.046252172	-	23.14353487	)	16.94656186	(	8.233611499	-	54.49715719	)	0.584
N-Acetylglucosamine	43.59892936	(	29.95685368	-	96.34014802	)	32.55597787	(	25.7916651	-	56.85775988	)	0.784

*statistically significant (p <0.05)

SCC: squamous cell carcinoma
